# Effectiveness of community health management for hypertensive patients under the medical alliance in China: a systematic review and meta-analysis

**DOI:** 10.3389/fpubh.2025.1460246

**Published:** 2025-06-02

**Authors:** Yi-Chun Gu, An-Qi Wang, Da He, Chang-Hai Tang

**Affiliations:** ^1^Shanghai Health Development Research Center (Shanghai Medical Information Center), Shanghai, China; ^2^School of Public Health, Dalian Medical University, Dalian, Liaoning, China; ^3^School of Nursing, Dalian University, Dalian, Liaoning, China

**Keywords:** integrated healthcare, medical alliance, community health management, hypertension, primary healthcare

## Abstract

**Objective:**

The prevalence of hypertension in China has been increasing, imposing a heavy burden on premature mortality and catastrophic healthcare expenditure. This study aims to evaluate the effectiveness of community health management on blood pressure control for hypertensive patients under the medical alliance, a model of integrated care in China.

**Methods:**

Following PRISMA guidelines, three English databases and three Chinese databases were searched from January 2010 to March 2024, with two reviewers involved in the screening, data extraction, quality assessment, and narrative synthesis with characteristics and interventions. Meta-analysis was performed with the value of Hedge’s g and 95% confidence interval (CI), using the random-effects model. Subgroup and meta-regression analysis were used to analyze the sources of heterogeneity. Egger’s test was applied to detect publication bias.

**Results:**

Twenty-five studies covering 7,727 hypertensive patients were included, with one of them assessed as low risk of bias. The pooled result showed that compared to conventional community health management, community health management of hypertension under the medical alliance had a more significant effect on reducing systolic blood pressure (Hedge’s g: −0.721, 95% CI: −0.890 to −0.552, *p* < 0.001) and diastolic blood pressure (Hedge’s g: −0.786, 95% CI: −1.002 to −0.571, *p* < 0.001). It could not be demonstrated that regional distribution, mean age of participants, sample size of the experimental group, sample size of the control group, duration of intervention, and publication year were the sources of heterogeneity. There was a significant publication bias in diastolic blood pressure.

**Conclusion:**

This study supports the effectiveness of community health management under the medical alliance in reducing blood pressure for hypertension. The guidance from general hospitals to community health centers, remote monitoring systems to expand the spatial scope of healthcare access and achieve information sharing, and two-way referral are efficient measures in the medical alliance.

## 1 Introduction

Hypertension, one of the leading causes of the increasing incidence of cardiovascular events and stroke, has been documented as a common chronic disease and a global public health issue (1). Previous studies have shown that hypertension contributes to 6% of the global burden of disease, affecting nearly one-third of the global population and leading to 7.7 million deaths annually (2, 3). With a growing aging population and a rising number of unhealthy lifestyles, the prevalence of hypertension in China has also been experiencing an increasing trend (4). According to *the Report on Cardiovascular Health and Diseases in China 2022*, individuals with hypertension have reached 245 million, and the total cost of hospitalization for hypertension was approximately $1.85 billion (5). Meanwhile, studies have shown that around 50% of hypertensive patients in China have not taken any medication, and around 80% of hypertension was uncontrolled, with significant differences between different geographical regions in the level of control and treatment of hypertension (6).

As in other countries, poor adherence to long-term medication and health management serves as the underlying cause of hypertension (7). In addition, inequalities in healthcare access disproportionately affect those with hypertension in lower socio-economic groups and further lead to health and economic inequalities (8). Accordingly, National Essential Public Health Services was launched by the Chinese central government in 2009, with the important objective of providing affordable screening and treatment for chronic diseases to promote equality in healthcare access. Community health centers, as government-run primary healthcare institutions for the prevention and control of non-communicable diseases, play a vital role in public health services such as hypertension management (9). Community health management is mainly provided by community health centers, and approximately 90% of hypertensive patients in China are under health management by urban and rural community health centers (10). The key tasks of community health management of hypertension are to inform about the dosage of medication and instructions on diet and exercise, to develop health records, and to provide health education as well as regular follow-up, which has been established as an important practice to avoid exacerbation and complications of hypertension (11). Community health management had desirable effects on antihypertensive medication use, blood pressure monitoring and equality of healthcare access (6). However, the hypertension control rate still has significant room for improvement by addressing the problem of shortage in the primary healthcare workforce, inadequate community health management and fragmentation in healthcare services (9, 10).

International experience suggests that integrated care is one of the essential pathways to integrating fragmented healthcare services, improving primary healthcare and enhancing health management for patients with chronic diseases (12, 13). In 2016, a collaborative report on Chinese medical and health system reform, released by the World Bank, the World Health Organization and the Chinese Government, provided eight recommendations centered on promoting the development of people-centered integrated care in China (14). In 2017, guidelines for constructing the medical alliance, as a major means of achieving people-centered integrated care in China, were issued by the Office of the State Council (15). The medical alliance is a collaborative alliance or medical group formed by different levels or types to integrate vertical and horizontal resources, combine medical information and provide continuity of service (16). Medical alliance is based on the principle of government-led integrated planning, on a grid basis, and then formed into a consortium according to the functions, positioning and levels of different medical institutions. Within the consortium, a human-oriented, patient-centered, whole chain of continuous medical services is formed. It can promote the construction of a mechanism of hierarchical diagnosis and treatment and division of labor through information sharing, resource sharing, talent sharing and corresponding management mechanisms, so as to provide patients with homogenized, high-quality and efficient medical services. In Chinese practice, the main mode of the medical alliance is the vertical integration of different levels of medical organization comprising general hospitals and community health centers, with the feature of shaping an orderly healthcare system using community-based primary healthcare, specialist-to-community technical guidance and two-way referrals (17, 18). As general hospitals in China have overwhelming medical resources and a much superior capacity for medical services than the community health centers, vertical integration of organizations is a prerequisite for achieving integrated care (14).

Trials conducted in multiple countries have proven the clear benefits of integrating community health workers into the vertical process of hypertension management (19). Within the medical alliance in China, effective coordination between general hospitals and community health centers can improve the shortage of primary healthcare workforce and inadequate community health management, and guarantee the continuity of care for hypertension through early identification, individualized healthcare plans, standardized health management, information sharing and referral procedures in the community, which has worked remarkably well in controlling blood pressure, minimizing the complications and premature mortality caused by ill-managed hypertension (9, 20). In the light of the fact that well-conducted systematic reviews and meta-analyses of randomized controlled trials provide the most valid research evidence on the effectiveness of interventions (21), it is, therefore, the aim of this meta-analysis to provide a comprehensive evaluation of the effectiveness of community health management on blood pressure control for hypertension under the medical alliance, and to help potentially achieving policy guidelines for hypertension control toward the provision of high-quality care.

## 2 Methods

### 2.1 Search strategy

Following the methodology of Cochrane systematic reviews and PRISMA guidelines (22), this study involved searching, evaluating, and combing articles published from January 2010 to March 2024, which were relevant to high blood pressure control, medical alliance, and community health management. Three English databases (PubMed, Web of Science, EBSCO) and three Chinese databases (China National Knowledge Infrastructure, WanFang Database, China Science and Technology Journal Database) were searched by using medical subject headings (MeSH) and broad derivatives, including hypertension, high blood pressure, medical alliance, medical association, medical community, medical cluster, medical consortium, medical group, medical combination, medical treatment combination, county medical community, county medical group, China, Chinese, and the full search strategy was shown in Supplementary material 1. References included in randomized controlled trials (RCTs) and gray literature were traced and hand-searched to identify potentially eligible studies, and authors were contacted for supplementary information where necessary. The study was registered in PROSPERO with CRD42021279005.

### 2.2 Including and excluding criteria

Studies were eligible for inclusion with the following criteria: (a) Baseline surveys were conducted in Mainland China. (b) Participants were diagnosed with hypertension according to *the Guidelines for Prevention and Treatment of Hypertension in China*. (c) Interventions were defined clearly, and the experimental groups intervened with community health management under the medical alliance, while the control groups received conventional community health management. (d) The intervention lasted for 3 months at least. (e) The baseline and endline information of systolic blood pressure (SBP) and diastolic blood pressure (DBP) for the participants in the experimental and control groups were provided separately.

Studies were excluded as follows: (a) Studies with unclear interventions and inadequate duration were excluded. (b) Participants with secondary hypertension, as well as other chronic diseases or other serious complications of hypertension, were not included. (c) Conference abstracts, editorials, correspondence and systematic reviews that were not RCTs or did not provide accurate outcome data were excluded. For potentially non-randomized controlled study, an initial appraisal is carried out by perusing the titles and abstracts. If a study is unambiguously identified as non-randomized, it is promptly excluded. When the study type cannot be determined from the title and abstract, the full text is then examined. Once a study is verified as non-randomized, it is removed from the inclusion scope.

### 2.3 Study selection

We allocated articles to two reviewers after duplicated articles exclusion by exporting to endnote software. Two reviewers conducted the screening of titles and abstracts with the study objectives, and judged whether an article was “included,” “excluded” or “inconclusive” depending on the criteria. A senior reviewer was consulted if there were inconsistent judgments between the first two reviewers for the same article. The Kappa consistency test, which showed an agreement between the two reviewers, was calculated with Excel and found to be 0.82.

### 2.4 Data extraction

Data extraction was developed by using a standardized information extraction form which was headed as follows: background information (e.g., author, year of publication, region conducting trials), participants (e.g., inclusion criteria, age of participants, gender distribution), interventions (e.g., sample size, intervention, duration), and outcomes (e.g., blood pressure values). Reviewers were trained to meet uniform norms for information extraction. Then, one reviewer extracted data with cross-checks from another reviewer, and disagreements were resolved by a senior reviewer to promote consensus during the data extraction.

### 2.5 Quality assessment

The quality of the included studies was completed independently by two reviewers according to Cochrane Collaboration’s tool for assessing risk of bias 5.1.0 with seven items (23). Each of these studies was rated as A (yes), B (unclear), or C (no) according to the assessment manual. Items considered being of low bias if they were all grade A. If one or more items were grade B/C, the bias was medium/high, and the quality was grade B/C. Evaluation results were validated, and disagreements were resolved with the support of a senior reviewer, and a kappa consistency test was 0.87 between the first two reviewers.

### 2.6 Data synthesis and analysis

The collected data was analyzed by Stata 18.0. Heterogeneity was determined by *Q*-test and *I^2^* value (significant heterogeneity when *p* < 0.1, *I^2^* > 50%) (24, 25). Random effects models were used for pooling when heterogeneity was significant, and otherwise, fixed effects models were used for analysis. Forest plots were drawn to compare the effect of community health management under the medical alliance and conventional community health management on blood pressure values for hypertension, with Hedge’s g and 95% confidence interval (CI) to indicate the pooled effect, statistically significant at 95% CI excluding 0. Hedge’s g = 0.2 is a small effect size, Hedge’s g = 0.5 is a medium effect size, and Hedge’s g = 0.8 is a large effect size (26). Subgroup analysis was conducted based on regional distribution (eastern, central, and western regions of China), mean age of participants (≤60 years old, >60 years old), experimental sample size (≤100, >100), control sample size (≤100, >100), duration of intervention (≤6 months, >6 months), publication year (2019 and before, 2020–2021, 2022 and beyond). Meta-regression was used to analyze sources of heterogeneity. Egger’s test was applied to detect the publication bias of the included articles, with *p* < 0.05 indicating the presence of publication bias.

## 3 Results

### 3.1 Study selection

**Figure 1:**
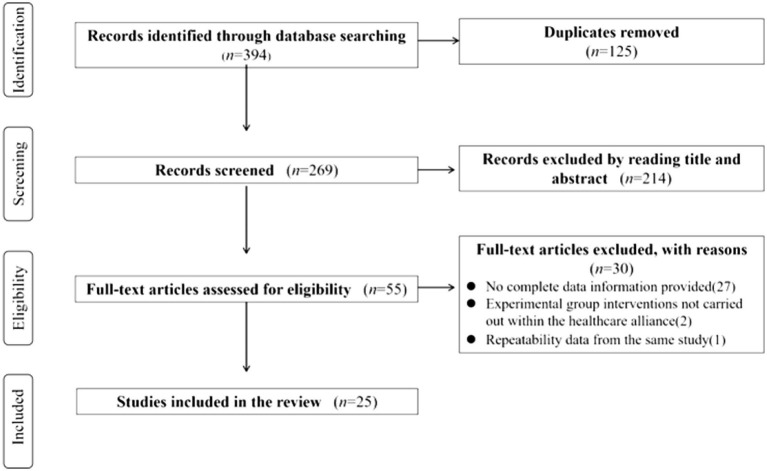
PRISMA flow diagram of the literature review process.

A total of 394 articles were retrieved across six electronic databases with a comprehensive search strategy. After excluding duplicates, the remaining 269 articles were to be screened for titles and abstracts. Then, 55 articles were identified for full-text review based on inclusion and exclusion criteria. After removing ineligible articles, 25 articles were included in the final selection for analysis. Figure 1 provides a detailed description of the selection process for the included studies using the PRISMA flow diagram.

### 3.2 Study characteristics

A total of 25 studies were included in the analysis (27–51). There were 4,063 and 3,664 hypertensive patients allocated to the experimental and control groups, respectively, and their mean age was 60.50 years. Sixteen of these studies were conducted in six provinces in eastern China, seven in four provinces in central China, and two in two provinces in western China. The average duration of intervention for participants was 11.40 months, with interventions largely after 2013 and publication year concentrating in 2017 or later. A description of the basic features of the included studies is given in Table 1.

**Table 1:** Characteristics of the included studies.

No	Author	Publication year	Mean age	Experimental sample size	Control sample size	Interventions		During of intervention (months)	Region
						Experiment	Control		
1	Wu Y (27)	2017	51.95	100	100	Conventional health management + Standardized management of medical alliance	Conventional health management	24	Guangdong
2	Zhong H T (28)	2017	57.16	500	200	Standardized management under the medical alliance	Conventional health management	5	Zhejiang
3	Cai M Z (29)	2017	62.81	117	111	Essential public health services and medical service under the medical alliance	Essential public health services + Medical service	30	Xinjiang
4	Shao S S (30)	2018	68.87	60	60	Hierarchical intervention under the medical alliance	Conventional intervention model for hypertension	6	Jiangxi
5	Zhang T (31)	2018	54.69	59	58	Chronic disease management under the medical alliance	Conventional health management	6	Jiangsu
6	Tang C (32)	2019	53.95	189	191	“Internet Plus” technology + “Hospital-family-community” three-way linkage hypertension management	Community-oriented Conventional chronic disease management model	6	Jiangsu
7	Yan M (33)	2020	49.50	54	54	“Internet Plus” technology + Resource sharing under the medical alliance	Conventional drug intervention and management	6	Guangdong
8	Sun G L (34)	2020	61.66	91	89	Conventional health management + Health education under the medical alliance	Conventional health management	6	Peking
9	Duan C C (35)	2020	54.93	60	60	Family doctor team intervention + primary hypertension management model involving nurse practitioners	Family doctor team intervention	12	Anhui
10	Yang J W (36)	2020	56.04	291	284	Conventional health management + Hypertension remote management platform under the medical alliance	Conventional health management	12	Peking
11	Qian L F (37)	2020	70.13	49	56	Health education under the medical alliance	Conventional health management	6	Anhui
12	Zhang X D (38)	2021	62.06	307	267	Management of “hypertension compliance centers” under the medical alliance	Conventional chronic disease management	6	Shandong
13	Mao L D (39)	2021	63.90	85	85	Hierarchical diagnosis and management under the medical alliance	Conventional health management	12	Guangdong
14	Liao Q J (40)	2021	65.22	80	80	Health education based on WeChat platform under the medical alliance	Conventional health education	12	Guangdong
15	Hu L H (41)	2021	58.06	350	350	Conventional diagnosis and management + medical alliance model	Conventional health management	24	Guangdong
16	Chen G X (42)	2021	69.17	147	72	Health management by chronic disease management teams of tertiary general hospitals in conjunction with community family doctors	Health management by community family doctors	24	Guangdong
17	Jin J (43)	2021	55.81	303	301	Hypertension management under the medical alliance	Conventional health management	6	Hunan
18	Li L (44)	2022	64.82	210	235	Health management of hypertension under the medical alliance collaboration	Conventional health management	6	Hubei
19	Ren L J (45)	2022	69.45	50	50	Conventional health management + Clinical pharmacists participating in community hypertension management under the medical alliance	Conventional health management	12	Jiangsu
20	Feng L L (46)	2022	54.90	600	600	Remote management platform under the medical alliance	Conventional health management	18	Shanghai
21	Liao Q J (47)	2023	61.77	100	100	Health management under the medical alliance	Conventional health management	10	Guangdong
22	He W H (48)	2023	55.93	30	30	Remote integrated management under the medical alliance	Conventional intervention for hypertension	12	Guangdong
23	Qin P (49)	2023	60.00	100	100	Conventional hypertension management model + medical alliance model	Conventional hypertension management model	6	Hunan
24	Lu X L (50)	2023	70.73	51	51	Conventional health management + Team-based chronic disease management under the medical alliance model	Conventional health management	12	Jiangxi
25	Zheng T R (51)	2023	58.91	80	80	Conventional health management + Grid city medical alliance model	Conventional health management	6	Sichuan

In addition, interventions of standardized community hypertension management under the medical alliance in the experimental group mainly covered the following: general hospitals to help community health centers improving capacity in screening and treatment of hypertension, forming multi-disciplinary teams, more individualized and efficient management in health monitoring, education and follow-up, building remote monitoring systems and improving timely two-way referrals. Conventional community hypertension management in control groups just comprised basic medication guidance, regular general health monitoring, follow-up visits and health education.

### 3.3 Quality assessment

Each of the included studies was critically assessed for the risk of bias using the Cochrane Collaboration’s tool, and the result is shown in Table 2. Only one study was assessed as low risk, the others were assessed as medium or high risk. Eleven studies reported randomized sequence generation, and only one reported allocation concealment methods in detail. All studies reported blinding of participants, personnel, and outcome assessment, or that measurements were not affected by blinding. All studies provided adequate information on complete outcome data, and all indicated a low risk of selective reporting bias. Only one study was free of other biases, and the others were all possible other biases.

**Table 2:** Risk assessment of included articles.

No.	Author	Random sequence generation	Allocation concealment	Blinding of participants and personnel	Blinding of outcome assessment	Complete outcome data	Selective reporting	Other sources of bias
1	Wu Y	A	B	A	A	A	A	B
2	Zhong H T	B	B	A	A	A	A	B
3	Cai M Z	C	C	A	A	A	A	B
4	Shao S S	A	B	A	A	A	A	B
5	Zhang T	A	C	A	A	A	A	B
6	Tang C	A	A	A	A	A	A	A
7	Yan M	A	B	A	A	A	A	B
8	Sun G L	C	C	A	A	A	A	B
9	Duan C C	C	B	A	A	A	A	B
10	Yang J W	A	B	A	A	A	A	B
11	Qian L F	C	C	A	A	A	A	B
12	Zhang X D	B	B	A	A	A	A	B
13	Mao L D	C	C	A	A	A	A	B
14	Liao Q J	C	C	A	A	A	A	B
15	Hu L H	A	B	A	A	A	A	B
16	Chen G X	C	C	A	A	A	A	B
17	Jin J	C	C	A	A	A	A	B
18	Li L	C	C	A	A	A	A	B
19	Ren L J	A	C	A	A	A	A	B
20	Feng L L	C	C	A	A	A	A	B
21	Liao Q J	A	C	A	A	A	A	B
22	He W H	A	C	A	A	A	A	B
23	Qin P	C	C	A	A	A	A	B
24	Lu X L	A	C	A	A	A	A	B
25	Zheng T R	C	C	A	A	A	A	B

### 3.4 Meta-analysis

#### 3.4.1 Meta-analysis of SBP

The fixed-effect model showed *I^2^* = 91.79%, *H^2^* = 12.17, Tau^2^ = 0.1621, Hedge’s g = −0.610 and the 95% CI was [−0.656, −0.563], *Q*-test showed *p* < 0.001, Considering the existence of heterogeneity, the random-effect model was analyzed by Der Simonian-Laird method.

**Figure 2:**
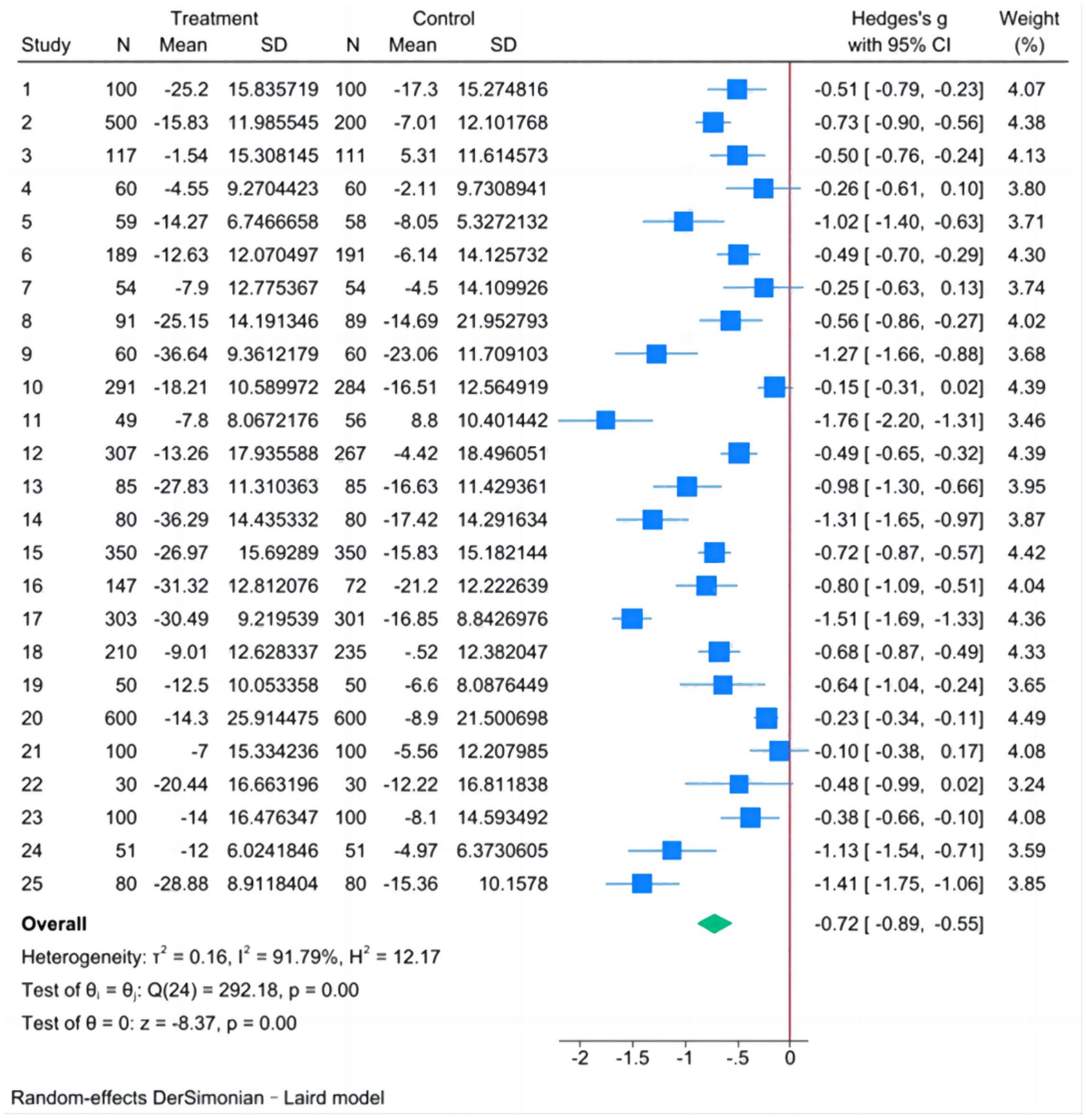
Forest plot of the difference in SBP decline between experimental and control groups.

The analysis results of the random-effect model in Figure 2 showed that the difference between the SBP decline in the experimental group and that in the control group was statistically significant, except for studies 4, 7, 10, 21, and 22. The pooled results showed that Hedge’s g = −0.721, 95% CI was [−0.890, −0.552], and the *Q*-test showed *p* < 0.001. Therefore, it can be assumed that the value of SBP in the experimental group had a greater degree of decline compared with that in the control group after the intervention.

#### 3.4.2 Meta-analysis of DBP

The fixed-effect model showed *I^2^* = 94.97%, *H^2^* = 19.88, Tau^2^ = 0.2716, Hedge’s g = −0.566 and the 95% CI was [−0.613, −0.520], *Q*-test showed *p* < 0.001, Considering the existence of heterogeneity, the random-effect model was analyzed by Der Simonian-Laird method.

**Figure 3:**
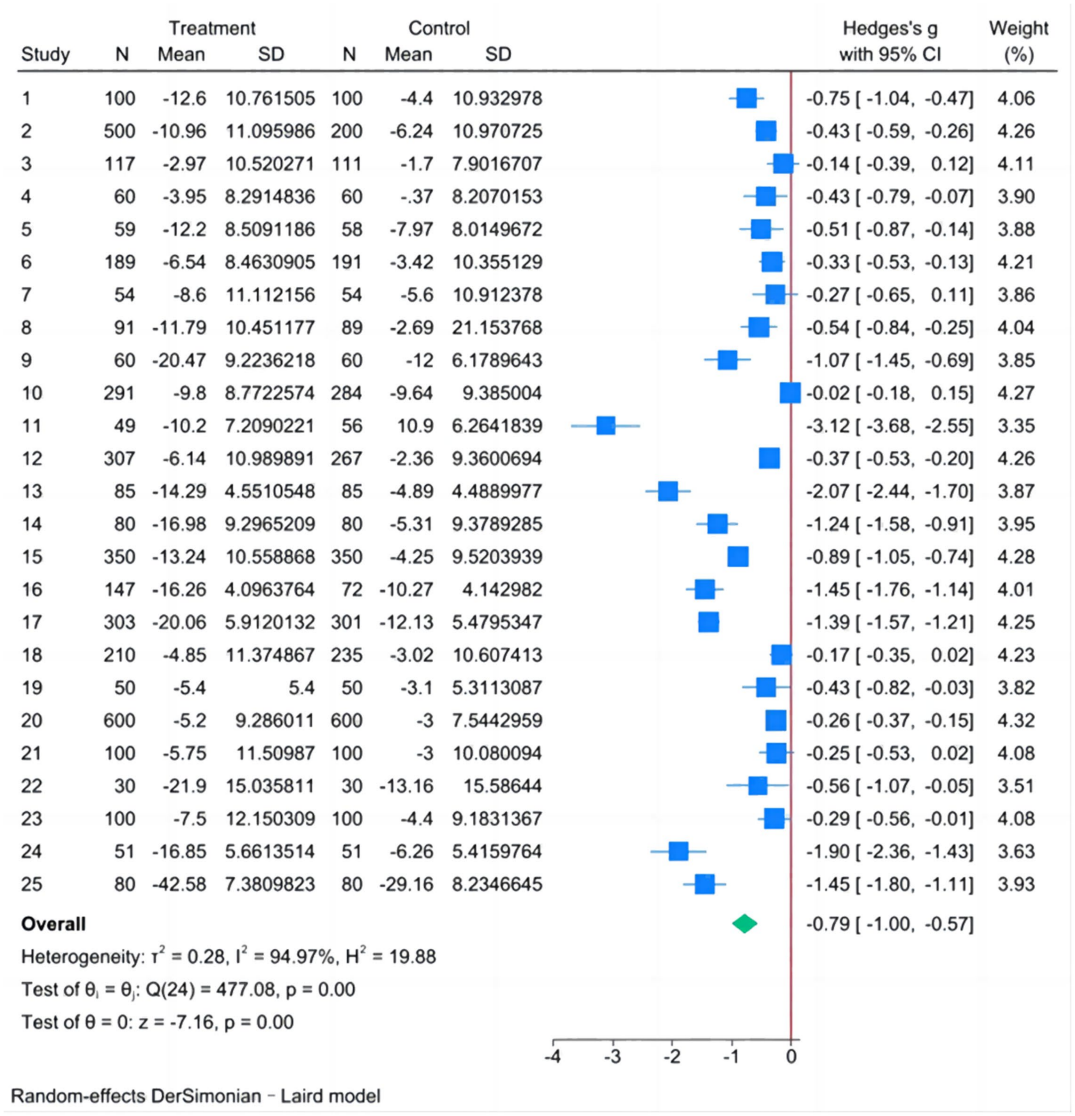
Forest plot of the difference in DBP decline between experimental and control groups.

The analysis results of the random-effect model in Figure 3 showed that the difference between the DBP decline in the experimental group and that in the control group was statistically significant, except for studies 3, 7, 10, 18, and 21. The pooled results showed that Hedge’s g = −0.786, 95% CI was [−1.002, −0.571], and the *Q*-test showed *p* < 0.001. Therefore, it can be assumed that the value of DBP in the experimental group had a greater degree of decline compared with that in the control group after the intervention.

### 3.5 Heterogeneity analysis

The *Q*-test results were statistically significant (*p* < 0.1) and the value of *I^2^* was greater than 50%, indicating that there was heterogeneity among the 25 included studies and that the sources of heterogeneity need to be further analyzed.

#### 3.5.1 Subgroup analysis

Subgroup analyses were conducted based on regional distribution, mean age of participants, sample size of the experimental group, sample size of the control group, duration of intervention, and publication year. Table 3 presents the results of the meta-analysis and heterogeneity tests for all subgroups. The results showed that in the two studies conducted in the western region, the differences in the decline of SBP and DBP between the experimental and control groups were not statistically significant (*p* > 0.05), and the results of the meta-analysis for all categories of the remaining subgroup variables were statistically significant (*p* < 0.05). As can be seen from the results of the *Q*-test for each category in the table, as well as the *I^2^* and *H* values, heterogeneity still existed in the various categories of subgroup variables (*p* < 0.1, *I^2^* > 50%, and *H* > 1.5) (52), and categorizing the included studies according to these factors did not eliminate heterogeneity. Therefore, these factors were not yet considered to be the sources of the heterogeneity. In addition, in the subgroups of control sample size and publication year, the difference in DBP decline between the experimental and control groups differed with different control sample sizes and different publication years.

**Table 3:** Subgroup analysis.

Blood pressure	Subgroup meta-analysis summary						Heterogeneity summary					Tests of group differences		
	Variable	No. of Studies	Hedges’s g	95% CI		*p* (Hedges’s g)	*df*	*Q*	*p* (*Q*)	*I^2^* (%)	*H*	*df*	*Q_b_*	*p* (*Q*_b_)
Systolic blood pressure	Regional distribution													
	Eastern	16	−0.578	−0.753	−0.403	<0.001	15	107.33	<0.001	86.02	2.68	2	3.71	0.157
	Central	7	−0.988	−1.515	−0.460	0.004	6	90.30	<0.001	93.36	3.88			
	Western	2	−0.947	−6.712	4.818	0.284	1	16.83	<0.001	94.06	4.10			
	Age													
	≤60 years	13	−0.698	−0.977	−0.419	<0.001	12	217.44	<0.001	94.48	4.26	1	0.07	0.791
	>60 years	12	−0.743	−1.028	−0.459	<0.001	11	73.07	<0.001	84.94	2.58			
	Experimental sample size													
	≤100	15	−0.795	−1.070	−0.520	<0.001	14	105.13	<0.001	86.68	2.74	1	0.89	0.345
	>100	10	−0.627	−0.898	−0.356	0.001	9	177.39	<0.001	94.93	4.44			
	Control sample size													
	≤100	16	−0.794	−1.049	−0.539	<0.001	15	105.32	<0.001	85.76	2.65	1	1.09	0.297
	>100	9	−0.609	−0.913	−0.306	0.002	8	174.80	<0.001	95.42	4.67			
	Duration of intervention													
	≤6 months	12	−0.785	−1.103	−0.467	<0.001	11	141.66	<0.001	92.23	3.59	1	0.56	0.456
	>6 months	13	−0.659	−0.904	−0.413	<0.001	12	114.62	<0.001	89.53	3.09			
	Publication year													
	2019 and before	6	−0.581	−0.822	−0.340	0.002	5	12.55	0.028	60.17	1.58	2	2.84	0.242
	2020–2021	11	−0.878	−1.226	−0.529	<0.001	10	176.91	<0.001	94.35	4.21			
	2022 and beyond	8	−0.614	−0.986	−0.243	0.006	7	67.13	<0.001	89.57	3.10			
	Overall	25	−0.721	−0.906	−0.536	<0.001	24	292.18	<0.001	91.79	3.49			
Diastolic blood pressure	Regional distribution													
	Eastern	16	−0.636	−0.850	−0.422	<0.001	15	211.78	<0.001	92.92	3.76	2	2.57	0.277
	Central	7	−1.165	−1.779	−0.551	<0.001	6	190.27	<0.001	96.85	5.63			
	Western	2	−0.789	−2.081	0.502	0.231	1	35.56	<0.001	97.19	5.96			
	Age													
	≤60 years	13	−0.628	−0.884	−0.372	<0.001	12	217.99	<0.001	94.50	4.26	1	2.08	0.149
	>60 years	12	−0.980	−1.384	−0.575	<0.001	11	256.35	<0.001	95.71	4.85			
	Experimental sample size													
	≤100	15	−0.975	−1.323	−0.627	<0.001	14	201.52	<0.001	93.05	3.79	1	3.66	0.056
	>100	10	−0.538	−0.819	−0.257	<0.001	9	232.46	<0.001	96.13	5.08			
	Control sample size													
	≤100	16	−1.005	−1.339	−0.671	<0.001	15	215.69	<0.001	93.05	3.79	1	6.45	0.011
	>100	9	−0.444	−0.720	−0.169	0.002	8	193.50	<0.001	95.87	4.92			
	Duration of intervention													
	≤6 months	12	−0.739	−1.057	−0.422	<0.001	11	223.07	<0.001	95.07	4.50	1	0.17	0.680
	>6 months	13	−0.833	−1.148	−0.518	<0.001	12	253.40	<0.001	95.26	4.60			
	Publication year													
	2019 and before	6	−0.417	−0.571	−0.263	<0.001	5	10.78	0.056	53.62	1.47	2	10.17	0.006
	2022–2021	11	−1.103	−1.507	−0.699	<0.001	10	302.89	<0.001	96.70	5.50			
	2022 and beyond	8	−0.632	−0.961	−0.303	<0.001	7	88.74	<0.001	92.11	3.56			
	Overall	25	−0.786	−1.002	−0.571	<0.001	24	477.08	<0.001	94.97	4.46			

#### 3.5.2 Meta-regression analysis

Tables 4, 5 present the results of meta-regression analysis for SBP and DPB, respectively, which showed that the *p*-values of the six factors, namely, regional distribution, mean age of the participants, sample size of the experimental group, sample size of the control group, duration of the intervention, and publication year, were all greater than 0.05, and all the 95% CI were included 0, which were not statistically significant, and it can be assumed that they were not the sources of heterogeneity.

**Table 4:** Meta-regression analysis of SBP.

Subgroup variables	Estimate	SE	*p*	95%CI
Regional distribution	−0.262	0.140	0.061	[−0.536, 0.012]
Age	0.025	0.180	0.890	[−0.328, 0.378]
Experimental sample size	−0.168	0.456	0.713	[−1.062, 0.727]
Control sample size	0.364	0.471	0.440	[−0.560, 1.287]
Duration of intervention	0.073	0.182	0.686	[−0.283, 0.430]
Publication year	0.032	0.120	0.792	[−0.203, 0.266]

**Table 5:** Meta-regression analysis of DBP.

Subgroup variables	Estimate	SE	*p*	95%CI
Regional distribution	−0.256	0.240	0.299	[−0.760, 0.247]
Age	−0.156	0.309	0.620	[−0.806, 0.494]
Experimental sample size	−0.497	0.788	0.536	[−2.152, 1.157]
Control sample size	0.987	0.814	0.241	[−0.723, 2.697]
Duration of intervention	−0.057	0.313	0.859	[−0.715, 0.601]
Publication year	0.023	0.206	0.911	[−0.409, 0.456]

### 3.6 Publication bias

Egger’s tests were used to elaborate on publication bias. With Egger’s Test *t* = −1.73 (*p* = 0.0965 > 0.05) for SBP, and Egger’s Test *t* = −3.26 (*p* = 0.0034 < 0.05) for DBP, the results showed that there was no significant publication bias in SBP, whereas there was a significant publication bias in DBP.

## 4 Discussion

### 4.1 The community-based management of hypertension under the medical alliance model demonstrates significant advantages

A total of 25 studies were included, in which the average intervention duration for participants was 11.40 months, incorporating 7,727 hypertensive patients from 12 provinces in China. In this meta-analysis, there is evidence that community health management under the medical alliance has a more significant effect on reducing blood pressure for hypertension than conventional community health management, which is consistent with previous studies that improved hypertension management in primary care settings benefited from the integrated healthcare delivery system (53). The distinct advantages of hypertension health management under the medical alliance, compared to conventional community health management, are specialists mentoring community health workers to improve medical capacity, forming multi-disciplinary teams, developing standardized health management plans, building remote monitoring systems and making two-way referrals to achieve the whole life-cycle management of hypertension, with a view on providing continuity, comprehensiveness, coordination and people-centered health services (54).

For one, specialists from general hospitals and community health workers are in a relationship of guidance and collaboration within the medical alliance. Specialists provide technical guidance on the community health management of hypertension in various ways, such as training sessions, on-site teaching and remote coaching (55), intending to improve community primary healthcare on hypertension in early screening, subsequent treatment and monitoring, and achieving homogenous management. Previous studies have also revealed that primary healthcare providers can improve blood pressure control with the guidance of specialists (10). Also, specialists and community health workers serve collectively as a multidisciplinary team, referred to in China as a family doctor team comprising general practitioners, hypertension specialists, community nurses and pharmacists (14). Combining professional skills with community health workers’ familiarity with the patients’ condition, specialists draw up individualized healthcare plans for hypertensive patients, taking into account medication, diet, exercise and psychological support. Glynn’s clinical trial designed to improve hypertension management in primary care settings also concluded that blood pressure control in patients gained from a multidisciplinary team with improved care coordination (56).

Second, standardized hypertension management is often considered as best practice. The goals of standardized community health management of hypertension, following the chronic disease management model under the medical alliance, are to strengthen the consciousness of hypertensive patients with a view to reversing the one-way management of patients by doctors and promoting active interaction with them for participation in blood pressure management in the community. Instead of simply informing patients of their medicine regimen and training healthy lifestyle, standardized community health management allows for real-time monitoring of hypertensive patients’ medication, exercise and diet with the help of internet-based remote technology or assignments for specialists, and instructs on self-monitoring of daily blood pressure to determine the existing health problems with no delay (57). Empirical evidence suggests that blood pressure control outcomes can be improved by self-health management and high-frequency blood pressure monitoring (58). In addition, a comprehensive assessment of the risk factors of hypertension-related disease is available for stratified management, which is supported by to development of individualized health records replacing the conventional ones.

Besides, the standardized community health management of hypertension has also further optimized the former pattern of health education and regular follow-up. Given that patients appreciate the authority of the specialist, it is possible to increase compliance with medical advice by involving the specialist in health education at community health centers. Also, one-to-one health consultations or small group health talks integrated with the patients’ specific situation enable to achieve the target of health education on promoting health literacy and self-health management behaviors. Furthermore, the format of follow-up has also shifted from home visits to various options for telephone, WeChat and home visits, increasing the frequency of follow-up visits and facilitating early monitoring of the health risk factors.

Third, remote monitoring systems and two-way referrals within the medical alliance further support the whole life-cycle management of hypertension. Remote monitoring systems enable community health workers and specialists to access the same information platform for efficient communication and continuous care of hypertension. It is particularly important when there are clinical complications of poor blood pressure control that cannot be dealt with by community health workers and specialists need to be consulted. Remote monitoring functions virtually extend access beyond the physical limits of health providers, improving service capabilities through remote consultation and assessment (58). Once the hypertensive patients are stabilized, the general hospitals transmit the patients’ basic information and rehabilitation plans down to the community health centers using the remote monitoring system, and monitor the patients’ recovery on a real-time. In case of abnormalities occurring, a referral can be made immediately to general hospitals for specialized treatment, with the operational process for two-way referrals (59).

### 4.2 Although regional variations exist, the related measures still hold reference value

A comprehensive analysis of central and western region studies shows that despite resource gaps compared to the east, the medical alliance model has achieved positive results in hypertension management. For instance, through two-way referral and primary-care physician training in the central-western region, the hypertension control rate improved significantly. This indicates that key aspects like tiered diagnosis and treatment and talent development can be effectively implemented in resource-constrained areas. Similarly, a western rural medical alliance used a mobile platform for remote blood pressure monitoring. Specialists adjusted treatment plans based on data, leading to increased patient awareness, treatment compliance, and reduced blood pressure. This demonstrates the model’s effectiveness in rural areas with limited resources. However, we recognize the significant internal variations in the central and western regions. Geographical, population, economic, and medical resource differences may affect model implementation. Although current data suggest applicability in some rural and underdeveloped areas, local conditions must be considered for wider application.

### 4.3 The follow-up period should be appropriately extended to ensure intervention effects

In the present study, given the relatively short follow-up periods in the 25 incorporated studies, with the majority spanning from 6 months to 1 year, accurately evaluating the long-term impacts of blood pressure control post-intervention proves challenging. From the extant research data, during the intervention phase, through the implementation of diverse intervention measures within the medical alliance model, such as enhanced patient education, optimized drug treatment management, and the establishment of an efficient hierarchical diagnosis and treatment mechanism, the blood pressure of hypertension patients was effectively regulated. Nevertheless, upon the cessation of intervention measures, the question of whether blood pressure can continuously maintain an optimal level remains indeterminate. For example, several of the included studies demonstrated that during the 3-month follow-up post-intervention, owing to the absence of continuous supervision and management, the hypertension-related unhealthy lifestyle habits of certain patients (such as a high-salt diet, insufficient physical activity, etc.) reemerged, leading to a certain degree of blood pressure elevation in some patients. Although it remained lower than the pre-intervention level, this phenomenon signals potential risks to the long-term sustainability of blood pressure control. Considering the aforementioned circumstances, future researches should significantly extend the follow-up period to comprehensively understand the long-term sustainability of blood pressure control post-intervention. Through long-term follow-up, we can better monitor the dynamic changes in patients’ blood pressure and analyze the factors contributing to blood pressure fluctuations, such as alterations in patient compliance, the influence of the continuity of medical services, and the perseverance of lifestyle modifications. This will furnish a crucial foundation for further refining the hypertension intervention strategies within the medical alliance model and formulating more long-term effective management plans.

### 4.4 Inter-group and intra-group differences were not consistent, but attention should still be paid to the practice implications of the intervention

Not all inter-group and intra-group differences in the subgroup analysis were statistically significant. We posit that the primary factors accounting for statistical significance within subgroups yet not between them are as follows:

First, Subgroup Homogeneity. Subgroups are defined by the characteristics of research objects. High within-subgroup homogeneity means interventions have consistent effects, showing significance at the subgroup level. However, inter-subgroup differences may not be crucial for intervention outcomes, making it hard to find statistical differences between subgroups. Second, Sample Size Variation. Sample sizes differ across subgroups. Small-sized subgroups have low statistical power, making it difficult to accurately identify between-subgroup differences. This reduces the likelihood of significant inter-subgroup comparisons. Third, Intervention Similarity. Intervention measures are mostly homogeneous. Similar strategies within subgroups lead to some effects, but subgroup differences are not substantial enough to cause significant changes in effect magnitude, resulting in non-significant inter-subgroup comparisons. Four, Clinical Equivalence. Even without statistical differences, subgroups may have equivalent intervention effects in clinical application. Subgroup divergences may not impact the clinical utility of intervention measures.

To mitigate heterogeneity in intervention measures, future investigations ought to refine their research designs. Creating a comprehensive, standardized protocol for the medical alliance model. Precise definitions of remote monitoring, expert involvement, and patient education can minimize result disparities. Improve Data Management: Using standardized tools for data collection. Control confounding factors by considering them during design and adjusting for them (e.g., genetic backgrounds, comorbidities) in data analysis using multivariate regression. This helps accurately assess the relationship between interventions and blood pressure control.

It’s worth noting that the western region’s subgroup analysis results were non-significant, which related to sample size, regional differences, and research quality: firstly, only two studies were included in the west, much fewer than 16 in the east and 7 in the central region. Small samples reduce statistical power, increase sampling error when analyzing blood pressure reduction, and may not accurately reflect the effect of community health management, resulting in non-significant results. Secondly, the west differs from the east and central in economic development, medical resources, and residents’ lifestyles. Weak medical infrastructure, shortage of staff, and low residents’ health awareness in the west restrict community health management and compliance, making the intervention effect less obvious and subgroup analysis non-significant. Thirdly, the overall included studies and those in the west may have low quality. Lack of randomization and allocation concealment information, and methodological weaknesses introduce biases, fail to control confounding factors, and mask the effect of community health management, leading to non-significant subgroup analysis results.

### 4.5 Bias in research requires adequate attention

Bias may stem from multiple factors. In our manuscript, the main source of bias is likely the limited literature retrieval scope, leading to restricted sample selection. The studies in this paper are limited to Chinese-language literature, causing sample constraints. Moreover, many studies lack comprehensive details on randomization and allocation concealment (only 11 studies reported random sequence generation, and only 1 study detailed the allocation concealment method), suggesting significant methodological flaws.

We recommend that, in the research design phase, standardize randomization and allocation concealment. Use validated techniques for random sequences to assign participants, ensuring group comparability and minimizing selection bias. Apply methods like the sealed-envelope or central randomization system to prevent premature group-assignment disclosure. During data collection and analysis, enforce strict quality control. Formulate meticulous implementation standards for medical alliance model intervention measures. Set clear criteria for remote monitoring, expert involvement, and patient education. Train relevant institutions and personnel to ensure consistent intervention implementation and reduce biases. For data collection management, unify blood pressure measurement device selection criteria, preferring accurate, clinically validated sphygmomanometers. Train measurement staff, calibrate devices regularly, create a comprehensive data collection plan, define variables, plan for missing data, and strengthen data quality control. Before data analysis, assess data distribution and structure to choose suitable statistical methods. Establish a data interpretation review mechanism. After initial analysis, have a multidisciplinary expert team review results to ensure objectivity and minimize subjective influence.

### 4.6 Setting and controlling SBP targets clearly and tightly is important

Recent clinical studies show strict SBP control reduces cardiovascular disease risk in hypertensive patients (60). For long-term prognosis, strict SBP control retards target-organ damage in hypertensive patients (60). We propose to incorporate the clarification of SBP targets as a pivotal aspect of the research agenda. Drawing upon the most recent hypertension treatment guidelines and research outcomes, and in consideration of the distinct characteristics of diverse patient groups within the medical alliance model, individualized and precisely defined SBP control targets will be established. For instance, in hypertensive patients with diabetes or cardiovascular disease risk factors, a more rigorous SBP target (e.g., <130 mmHg) will be prescribed; for general hypertensive patients, the standard target range recommended by the guidelines will be adopted. By comparing the impact of intervention strategies within the medical alliance under varying SBP targets, more targeted clinical guidance can be furnished.

These specific processes described above are consistent with the integrated information platform, self-health record management and others involved in the integrated healthcare system (61–63). In addition, this study carries a few implications for policy and practice in community health management of hypertension under the medical alliance.

Policy-makers play a pivotal role in hypertension community-based health management within the medical alliance. To enhance the efficacy and service quality, the following recommendations are proposed: first, clarifying duties and implementing benefit-sharing mechanisms. It is essential to delineate the respective roles of general hospitals and community health centers precisely. General hospitals should be responsible for managing complex cases, providing technical guidance, and conducting staff training. Community health centers, on the other hand, should focus on the day-to-day care of patients. Moreover, a well-designed benefit-sharing policy for tiered diagnosis and treatment cooperation should be established to ensure the seamless operation of the medical alliance. Second, strengthening policy support and resource allocation. In terms of financial resources, it is recommended that a special fund be set up for the improvement of hypertension management infrastructure in community health centers and the training of relevant staff. Regarding human resources, appropriate incentives should be provided to attract talent, strengthen the workforce, and explore management mechanisms that are more suitable for the local context. Third, improving supervision and evaluation systems. A comprehensive supervision system with rigorous quality standards should be constructed. Regular monitoring of the cooperation between hospitals and community health centers is necessary. An evaluation index system covering aspects such as blood pressure control and patient satisfaction should be developed. Based on the assessment results, policies should be adjusted to optimize the management model.

## 5 Limitations

Several limitations are worthy of attention: firstly, although the search for accessible database resources and references was carried out wherever possible, the included studies were published in Chinese journals, which may limit the generalizability of our findings. In subsequent research, our team intends to actively collect data on hypertension interventions under similar medical alliance models in other countries and regions. Aiming to elucidate the commonalities and differences of the medical alliance model among diverse populations and within different medical systems. Besides, considering that cultural disparities in different countries and regions may influence patients’ acceptance and compliance with intervention measures, we will conduct cultural adaptability research. Analyzing patients’ perceptions and feedback regarding remote monitoring, patient education methods, and the hierarchical diagnosis and treatment process in different cultural contexts and exploring how to make cultural adaptability adjustments to the medical alliance model to enhance its promotional effect among diverse populations. Which will verify and expand the applicability of the research results and comprehensively evaluate the effectiveness and feasibility of the medical alliance model on a global scale, providing more widely applicable experience and strategies for global hypertension management. Second, the included studies were assessed for inferior quality, with some methodological weaknesses, such as missing clear information on randomization and allocation concealment, which was still unsuccessful despite efforts to contact the authors. Thirdly, this meta-analysis found significant heterogeneity principally about systematic evaluations combining studies of clinical and methodological diversity, with differences in the different strategies of interventions. Thus, heterogeneity of results was expected, random-effects models were employed for pooling the results of the included studies, and sources of heterogeneity were attempted but not yet found. Fourth, limited by data integrity, research heterogeneity, and resources, we could not analyze diverse intervention measures. We’ll reach out to original research teams for more data, then use qualitative or quantitative methods to compare measure effects and develop a more effective hypertension intervention program.

## 6 Conclusion

The evidence from this study supports the effectiveness of community health management under the medical alliance in reducing blood pressure for hypertension. The standardized community management of hypertension with the guidance of general hospitals contains rigorous medication and blood pressure monitoring, stratified health management, effective health education, diversified and high-frequency follow-up visits. Remote monitoring systems have been established to achieve information sharing, expand the spatial scope of healthcare access, and tele-teaching between specialists and community health workers, together with on-site teaching and offline training, making it more likely for hypertensive patients to be provided with high-quality and individualized health management. In addition, developing multidisciplinary teams and two-way referrals between general hospitals and community health centers provide comprehensive and continuous care for hypertensive patients. Meanwhile, it is important to note that research bias can cause the stability and applicability of research conclusions to be reduced. Researchers are needed to pay attention to the direction of cutting-edge research, and conduct research design, data collection, organization and analysis in a scientific and rational manner based on key indicators and data as much as possible. In the implementation of community management of hypertension under the medical alliance model in different geographic regions and economic and cultural environments, it is necessary to tailor the key techniques and methods to local realities in order to make more effective interventions.
