# Supplementary material for: Effectiveness of community health management for hypertensive patients under the medical alliance in China: a systematic review and meta-analysis
Source: Front Public Health. 2025 Jun 2;13:1460246. doi: 10.3389/fpubh.2025.1460246 (PMC12171210; doi:10.3389/fpubh.2025.1460246)
Supplement: Supplementary file 1 [file Table_1.docx]

**PubMed:**

(((((((hypertension[MeSH Terms]) OR (blood pressure, high[Title/Abstract])) OR (blood pressures, high[Title/Abstract])) OR (high blood pressure[Title/Abstract])) OR (high blood pressures[Title/Abstract])) AND (((((((((((medical association[MeSH Terms]) OR (medical alliance[Title/Abstract])) OR (medical consortium [Title/Abstract])) OR (medical cluster[Title/Abstract])) OR (medical community [Title/Abstract])) OR (medical group[Title/Abstract])) OR (medical complex [Title/Abstract])) OR (medical combination[Title/Abstract])) OR (medical treatment combination[Title/Abstract])) OR (county medical community[Title/Abstract])) OR (county medical group[Title/Abstract]))) AND ((((China[MeSH Terms]) OR (People's Republic of China[Title/Abstract])) OR (Mainland China[Title/Abstract])) OR (Chinese[Title/Abstract]))) AND (("2010/01/01"[Date - Publication] : "2024/03/01" [Date - Publication]))

**Web of Science：**

① ((((TI=(hypertension)) OR TI=(blood pressure, high)) OR TI=(blood pressures, high)) OR TI=(high blood pressure)) OR TI=(high blood pressures) and Preprint Citation Index (Exclude – Database)

② ((((((((((TI=(medical association)) OR TI=(medical alliance)) OR TI=(medical consortium)) OR TI=(medical cluster)) OR TI=(medical community)) OR TI=(medical group)) OR TI=(medical complex)) OR TI=(medical combination)) OR TI=(medical treatment combination)) OR TI=(county medical community)) OR TI=(county medical group) and Preprint Citation Index (Exclude – Database)

③ (((TI=(China)) OR TI=(People's Republic of China)) OR TI=(Mainland China)) OR TI=(Chinese) and Preprint Citation Index (Exclude – Database)

④ PY=(2010-2024) and Preprint Citation Index (Exclude – Database)

① AND② AND③ AND④ and Preprint Citation Index (Exclude – Database)

**EBSCO：**

① TX hypertension OR TX blood pressure, high OR TX blood pressures, high OR TX high blood pressure OR TX high blood pressures

② TX medical association OR TX medical alliance OR TX medical consortium OR TX medical cluster OR TX medical community OR TX medical group OR TX medical complex OR TX medical combination OR TX medical treatment combination OR TX county medical community OR TX county medical group

③ TX China OR TX People's Republic of China OR TX Mainland China OR TX Chinese

④ Date of publication 20100101-20240301

① AND② AND③ AND④

**China National Knowledge Infrastructure:**

(hypertension[Topic]) AND (medical alliance[Topic]) AND (2010-01-01--2024-03-01 [Date of publication] )

**WanFang Database:**

(hypertension[Title/Keywords]) AND (medical alliance [Title/Keywords]) AND (2010-2024 [Publication year] )

**China Science and Technology Journal Database:**

(hypertension[Title/Keywords]) AND (medical alliance [Title/Keywords]) AND (2010-2024 [Publication year] )
